# Flavonoid-Mediated Sex-Specific Fluorescence in Silkworm Cocoons: A Strain-Restricted Trait

**DOI:** 10.3390/insects17050509

**Published:** 2026-05-17

**Authors:** Mingfa Ling, Rui Yang, Yalei Wang, Xiangbiao Liu, Yu Guo, Heying Qian, Huiduo Guo

**Affiliations:** 1 Jiangsu Key Laboratory of Sericultural and Animal Biotechnology, School of Biotechnology, Jiangsu University of Science and Technology, Zhenjiang 212100, China; 2 Key Laboratory of Silkworm and Mulberry Genetic Improvement, Ministry of Agriculture and Rural Affairs, Sericultural Scientific Research Center, Chinese Academy of Agricultural Sciences, Zhenjiang 212100, China

**Keywords:** silkworm, fluorescent cocoons, metabolomics, flavonoids, sex-dependent

## Abstract

Flavonoids have been implicated in the formation of sex-discriminating fluorescent silkworm cocoons. However, their specific composition in these cocoons remain unexplored. In this study, flavonoid-targeted metabolomics was employed to profile the flavonoid composition of a sex-dependent fluorescent silkworm cocoon strain. Our results revealed comparable total flavonoid content between the violet and yellow fluorescent cocoons, but markedly different flavonoid composition, particularly in a subclass of flavonols. Moreover, four differential flavonoids, baimaside, luteolin-3′,7-di-O-glucoside, quercetin 3,7-diglucoside, and spiraeoside, were identified, among which quercetin 3,7-diglucoside, the most abundant, with levels exceeding the others by an order of magnitude, emerged as the most promising biomarker for the sex-dependent fluorescent phenotypes. These findings pave the way for future investigations into the molecular basis of sex-dependent fluorescent cocoon formation and support the breeding of sex-discriminating fluorescent cocoons for high-quality silk production.

## Simple Summary

Flavonoids are involved in the formation of silkworm cocoons that exhibit sex-dependent fluorescence, but their specific composition has been unclear. Using targeted metabolomics, we compared a sex-dependent fluorescent strain. The results showed that total flavonoid content was similar between violet (female) and yellow (male) cocoons, yet the composition differed markedly, especially in subclass flavonols. Four differential flavonoids were identified. Among them, quercetin 3,7-diglucoside was the most abundant and emerged as the most promising biomarker. Our findings provide a foundation for understanding the molecular basis of sex-dependent fluorescence and for breeding sex-discriminating cocoons for high-quality silk.

## 1. Introduction

Sericulture has a long-standing history and occupies a significant position in the national economies of many countries, particularly in developing regions of Africa and Asia [1,2,3]. Domestic silkworm (*Bombyx mori*) cocoons are widely used for various purposes, including textiles, medical applications, and cosmetics [4,5,6,7]. Previous studies have reported that cocoons of certain silkworm strains exhibit sex-dependent fluorescence under ultraviolet light [8,9,10]. Notably, cocoons emitting yellow fluorescence are predominantly male and exhibit superior textile properties, whereas violet-fluorescent ones are more frequent among females [8,9]. Given that male cocoons are generally superior in quality to their female counterparts, this distinctive trait has prompted efforts to breed strains with high fluorescence sex-discrimination accuracy, aiming to improve cocoon quality and production efficiency. However, the underlying mechanisms governing fluorescence formation remain poorly understood, which has hindered the breeding and practical utilization of such sex-discriminating strains. Therefore, elucidating the differential mechanisms underlying sex-specific cocoon fluorescence is of great significance for overcoming current breeding bottlenecks and ultimately enhancing both the quality and efficiency of silkworm cocoon production.

The domestic silkworm is a monophagous insect that feeds exclusively on mulberry leaves, which contain considerable amounts of flavonoids [11,12,13]. Flavonoids are a group of polyphenolic compounds produced in plants as secondary metabolites, which have received increasing research interest in recent years, because of their biological antioxidant activity and favorable biochemical effects on many diseases [14,15,16,17,18,19]. Also, flavonoids, particularly quercetin and its metabolites, serve as key determinants of pigmentation in many organisms, including the green coloration of silkworm cocoons [20,21,22,23,24]. Multiple genes involved in flavonoid metabolism and transport have been demonstrated to contribute to color cocoon formation, namely, quercetin 5-O-glucosyltransferase, glycoside hydrolase family 1 group G 5 (GH1G5), pyrroline-5-carboxylate reductase (P5CR), and a sugar transporter gene cluster [20,21,22,25,26]. Moreover, previous studies have identified flavonoids as key contributors to the formation of fluorescent cocoons [10,27,28,29]. In fact, natural flavonoids with broad-spectrum pharmacological activities can exhibit unique dual-emission fluorescence properties [30]. However, flavonoids present in silkworm tissues differ from those found in mulberry leaves, indicating host-mediated metabolic transformation and posing challenges for further study [10,29]. Using thin-layer chromatography (TLC), Zhang Yuqing et al. identified flavonoids as the main components of fluorescent cocoons in a sex-dependent fluorescent silkworm cocoon strain [10]. In a follow-up study, they further identified many proteins as potential binding candidates for fluorescent pigments in silkworm tissues [29]. In addition, Tejas S Kusurkar et al. successfully extracted fluorophores from the fluorescent silk cocoon membrane and identified them as primarily quercetin derivatives [27]. Recently, a study identified pentosyl flavonoid glycoside as a male-enriched pigment [31]. While these studies suggested that flavonoids are the primary contributors to fluorescent cocoon formation, the specific composition of these fluorescent substances remains unknown.

In this study, we employed flavonoid-targeted metabolomics to profile flavonoids in a sex-dependent fluorescent silkworm cocoon strain. Marked differences in flavonoid profiles were observed between the violet and yellow fluorescent cocoons, despite comparable total flavonoid content. Notably, quercetin 3,7-diglucoside, the most abundant flavonoid by an order of magnitude, emerged as a potential key biomarker for the sex-dependent fluorescent phenotype. This study thus provides a foundation for further investigation into the molecular basis of sex-dependent fluorescent cocoon formation in silkworms and supports the breeding of sex-discriminating fluorescent cocoons to produce high-quality silk.

## 2. Materials and Methods

### 2.1. Silkworm and Samples

The silkworm strain named “Xiangqing” was provided by the Sericultural Research Institute, Chinese Academy of Agricultural Sciences (Zhenjiang, China). Through selective breeding, this strain has acquired a stable and highly accurate sex-dependent fluorescence trait in the past 2–3 years, providing a valuable model for elucidating the mechanisms of fluorescent cocoon formation in silkworms. Larvae were reared on fresh leaves of mulberry under controlled conditions with a stable temperature of 25 °C, relative humidity of 75–85%, and natural light. After pupation, the cocoons were harvested and examined under UV light at 365 nm for fluorescence characterization. Based on their fluorescence phenotypes, cocoons exhibiting violet fluorescence (female) were assigned to the violet group, while those displaying yellow fluorescence (male) were designated as the yellow group.

### 2.2. Determination of Total Flavonoids

Total flavonoid contents of cocoons were determined using a commercial assay kit (G0118W, Suzhou Grace Biotechnology Co., Ltd., Suzhou, China) following the manufacturer’s instructions. Briefly, cocoons were cut into small pieces with a diameter of about 0.2–0.5 cm and 0.2 g cocoon shell samples were transferred to an EP tube, extracted with 4 mL of 60% ethanol under ultrasonic treatment for 15 min and oscillation at 60 °C for 4 h, and then centrifuged at 12,000 rpm for 5 min at room temperature. The resulting supernatants were collected for subsequent analysis. Following the kit protocol, the appropriate reagents were added, mixed thoroughly, and the mixture was allowed to stand at room temperature for 15 min. Absorbance was measured at 510 nm. Each sample was analyzed in four biological replicates, each with three technical replicates.

### 2.3. Metabolomic Analysis

#### 2.3.1. Chemicals and Reagents

HPLC-grade acetonitrile and methanol were obtained from Merck (Darmstadt, Germany), while formic acid was sourced from Sigma-Aldrich. Ultrapure water (MilliQ, Millipore, Bradford, MA, USA) was used throughout all experiments. All flavonoid standards were purchased from MedChemExpress (MCE, Shanghai, China). Individual stock solutions were prepared by dissolving each standard in 70% methanol to a final concentration of 10 mmol/L and stored at −20 °C. Prior to analysis, these stock solutions were appropriately diluted with 70% methanol to prepare working standard solutions.

#### 2.3.2. Sample Preparation and Extraction

Cocoon samples were freeze-dried and subsequently ground into a fine powder using a grinder (30 Hz, 1.5 min). For extraction, 20 mg of the powder was accurately weighed and mixed with 0.5 mL of 70% methanol containing 20 μL of internal standard (10 mg/L). The mixture was subjected to ultrasonic extraction for 30 min, followed by centrifugation at 12,000× *g* for 5 min at 4 °C. The resulting supernatant was carefully collected and filtered through a 0.22 μm membrane filter prior to LC-MS/MS analysis. The quality control samples were prepared by mixing an equal aliquot of the supernatant of the samples. Each group contained four pooled samples, with 3 cocoons per sample, and each sample was analyzed once by LC-MS/MS. Quality control samples were injected during the analysis to verify stability and accuracy.

#### 2.3.3. LC-MS/MS Analysis

Chromatographic separation was performed on an ExionLC™ AD UPLC system coupled with a QTRAP^®^ 6500+ mass spectrometer (Sciex, Framingham, MA, USA). Separation was achieved on a Waters ACQUITY UPLC HSS T3 C18 column (100 mm × 2.1 mm, 1.8 μm) maintained at 40 °C. The mobile phase consisted of water (A) and acetonitrile (B), both containing 0.05% formic acid. The gradient elution was programmed as follows: 0–1 min, 10–20% B; 1–9 min, 20–70% B; 9–12.5 min, 70–95% B; 12.5–13.5 min, 95% B; 13.5–13.6 min, 95–10% B; 13.6–16 min, 10% B. The flow rate was set at 0.35 mL/min, and the injection volume was 2 μL. Mass spectrometric detection was carried out using an ESI source operating in both positive and negative ion modes, controlled by Analyst 1.6.3 software (Sciex). The source parameters were set as follows: ion spray voltage at 5500 V (positive) and −4500 V (negative), source temperature at 550 °C, and curtain gas at 35 psi. Flavonoids were quantified using scheduled multiple reaction monitoring (MRM) mode. Compound-specific parameters, including declustering potential and collision energy, were optimized for each MRM transition. Data processing and metabolite quantification were performed using Multiquant 3.0.3 software (Sciex).

### 2.4. Statistical Analysis

Principal component analysis (PCA), orthogonal partial least squares discriminant analysis (OPLS-DA), and volcano plot analysis were performed on the MetWare cloud platform (http://www.metware.cn/, accessed on 20 January 2026). Flavonoid levels were measured by using SPSS 19.0 (IBM SPSS, Chicago, IL, USA). Data were presented as means ± SEM. *p*-values were calculated using a two-tailed unpaired Student’s *t*-test related to the indicated group, and *p* < 0.05 was considered statistically significant.

## 3. Results

### 3.1. Fluorescence Characteristics and Total Flavonoid Content of Sex-Dependent Fluorescent Silkworm Cocoons

**Figure 1:**
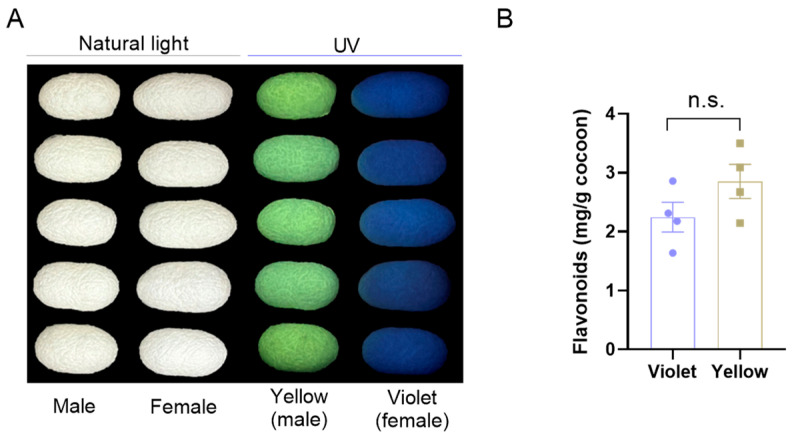
The content of total flavonoids in sex-dependent fluorescent silkworm cocoons. (**A**) Photographs of sex-dependent fluorescent silkworm cocoons under UV and natural light. (**B**) The levels of total flavonoids in sex-dependent fluorescent silkworm cocoons. Data are mean ± SEM. n.s. indicates no significant difference (*p* ≥ 0.05, two-tailed unpaired Student’s *t*-test).

In the present study, a sex-dependent fluorescent silkworm cocoon strain named “Xiangqing” was used to study the profiles of flavonoids in the cocoons. As shown in Figure 1A, male cocoons exhibited bright yellow fluorescence under UV light, whereas female cocoons displayed violet fluorescence, in contrast to their uniformly white appearance under natural light. Moreover, the total flavonoids in these cocoons were measured. The results showed comparable total flavonoid contents between violet and yellow fluorescent cocoons, suggesting that specific flavonoid components may be involved in the formation of the fluorescence phenotype.

### 3.2. Flavonoid Composition and Content in Sex-Dependent Fluorescent Silkworm Cocoons

The targeted metabolomics on flavonoids by LC-MS/MS identified a total number of 152 flavonoid species. Based on the oxidation degree of the central heterocycle, these flavonoids were then classified into seven subclasses: flavones, isoflavones, anthocyanidins, flavonols, flavanones, flavanols, and chalcones (Table 1). The results showed that most of the flavonoids are flavones, flavonols, and other types of flavonoids, accounting for 18.42% (28/152), 21.05% (32/152), and 31.58% (48/152), respectively. Consistent with their numerical abundance, the contents of these flavonoids also constituted a large majority of the total flavonoid content in both violet and yellow fluorescent silkworm cocoons. Moreover, the flavonol content in yellow fluorescent cocoons was approximately twice as high as that in violet cocoons, whereas the content of other flavonoids was approximately two-fold lower. These compositional differences may be associated with the distinct fluorescent phenotypes of the cocoons.

### 3.3. Dimensionality Reduction and Visualization of Flavonoid Data Based on PCA and OPLS-DA Models

**Figure 2:**
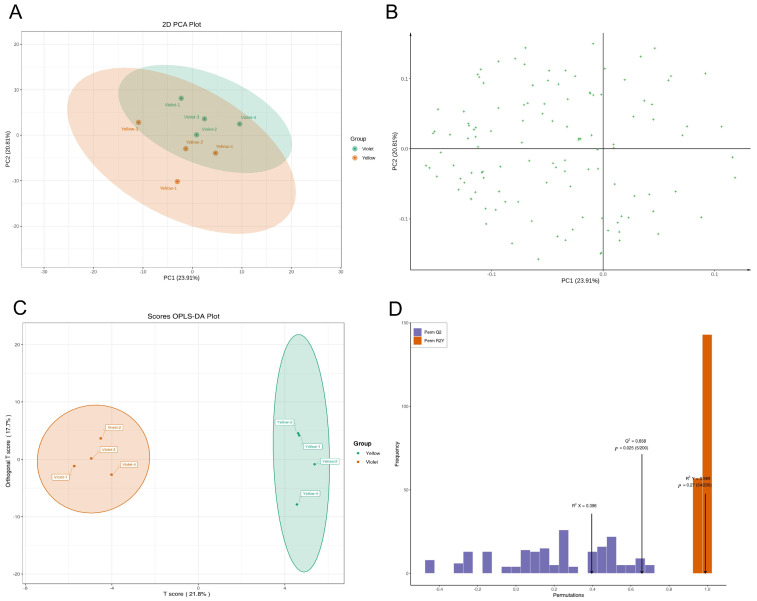
The PCA and OPLS-DA models of flavonoids in sex-dependent fluorescent silkworm cocoons. (**A**) Score plot of PCA model. (**B**) Loading scatter plot of PCA model. (**C**) Score plot of OPLS-DA model. (**D**) Permutation tests of the OPLS-DA model.

To understand the overall profile and patterns of flavonoid metabolism in the sex-dependent fluorescent silkworm cocoons, principal component analysis (PCA) and orthogonal partial least squares discriminant analysis (OPLS-DA) were performed. The results showed that sex-dependent fluorescent silkworm cocoon samples can be clustered into two groups in the PCA score plot (Figure 2A). However, the intra-group cohesion and inter-group classification clarity were still insufficient, according to the scatter plot of the PCA model with overlapping clusters (Figure 2B). Therefore, a supervised OPLS-DA model was used for further analysis. The OPLS-DA score plot revealed clear separation in the sex-dependent fluorescent silkworm cocoons, with principal component 1 (PC1) accounting for 21.8% of the total variance, and PC2 explaining 17.7% (Figure 2C). The OPLS-DA model exhibited good predictive ability (Q^2^ = 0.658, *p* = 0.025, with 200 permutation tests), indicating that the model could reliably capture the flavonoid metabolic differences between the two groups. Although the relatively high R^2^Y value (0.989, *p* = 0.27) suggested potential overfitting, the significance of Q^2^ and the model’s satisfactory predictive performance supported the subsequent screening of differential flavonoids (Figure 2D). Accordingly, a stricter set of criteria was applied in the subsequent screening of differential flavonoids to enhance the reliability of the results.

### 3.4. Screening of Differentially Accumulated Flavonoids

**Figure 3:**
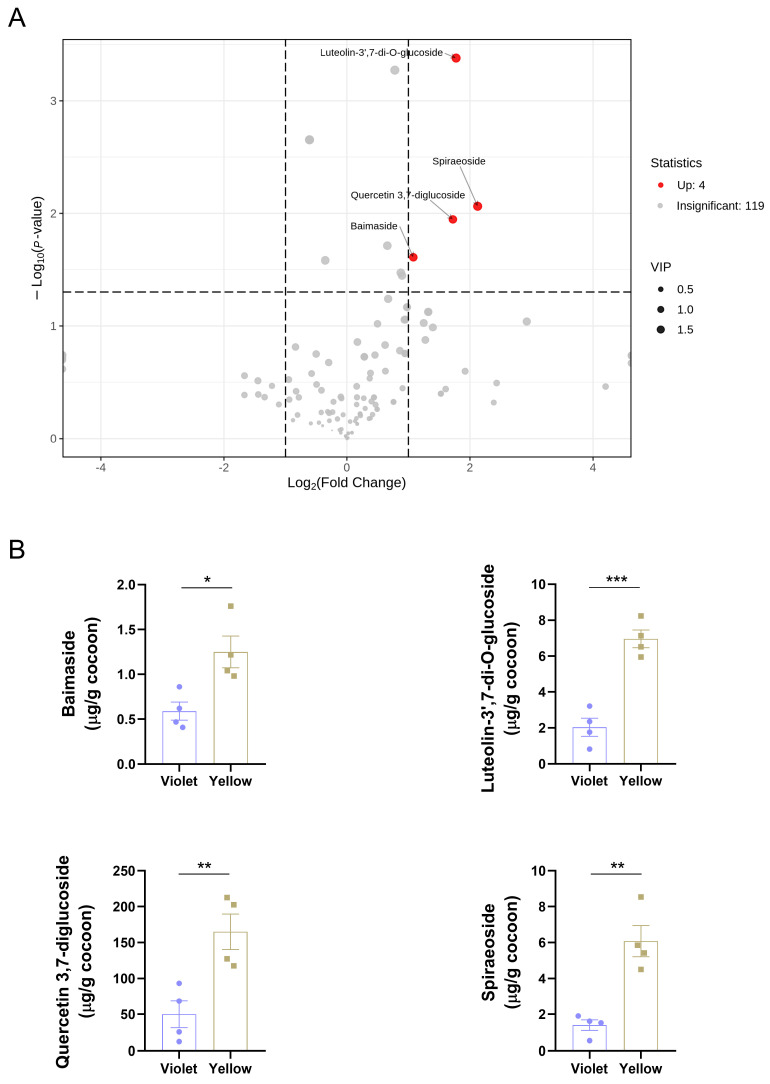
Screening of differential flavonoids. (**A**) Volcano plot with fold change, *p*-value, and VIP. (**B**) Contents of differential flavonoids. Data were presented as means ± SEM. *p*-values were calculated using a two-tailed unpaired Student’s *t*-test; *p* < 0.05 was considered statistically significant. * *p* < 0.05, ** *p* < 0.01, and *** *p* < 0.001.

Based on the stringent criteria of VIP > 1, fold change > 2 or < 0.5, and *p* < 0.05, four differential flavonoids, baimaside, luteolin-3′,7-di-O-glucoside, quercetin 3,7-diglucoside, and spiraeoside, were identified (Figure 3A). The bar chart further illustrated their respective contents in the cocoons. Notably, quercetin 3,7-diglucoside exhibited the highest abundance among both the four differential flavonoids and all detected flavonoids, with levels exceeding the others by approximately an order of magnitude (Figure 3B). This pronounced accumulation suggested that the metabolic processes involving quercetin may contribute to the formation of the distinct fluorescent phenotypes observed in these cocoons.

## 4. Discussion

In the present study, we aimed to investigate the flavonoid profiles in a sex-dependent fluorescent silkworm cocoon strain, characterized by bright yellow fluorescence in males and violet fluorescence in females under UV light. Marked differences were observed in the flavonoid profiles in the cocoons, with a set of candidate biomarkers emerging as potentially associated with this sex-dependent fluorescent phenotype. It was well known that many organisms in nature can exhibit autofluorescence, including jellyfish, butterflies, silkworms, wasps, and others [9,32,33,34]. In the case of domestic silkworm (*Bombyx mori*) cocoons, many studies have reported that they emitted fluorescence of various colors under UV light, a phenomenon observed to be sex-dependent in certain silkworm strains [8,9,10,29]. Similarly, during our breeding process of silkworm strains, we observed that the cocoons of the “Xiangqing” strain exhibited violet and yellow fluorescence under UV light, and those emitting violet fluorescence were mostly females, while those emitting yellow fluorescence were mostly males. This characteristic provides an opportunity for us to develop high-quality silk based on sex-dependent fluorescent cocoons, as existing methods, whether manual sorting, machine-based classification, or feeding silkworms with fluorescent carbon dots, are both time-consuming and material-intensive [35,36,37]. Although the silkworm strain we have bred has demonstrated a satisfactory sex-discrimination rate over the past 2–3 years, elucidating the mechanism governing fluorescent cocoon formation remains one of our primary objectives, as it may serve as the foundation and guarantee for maintaining this high sex-discrimination accuracy. Previous studies have indicated that flavonoids are key factors in cocoon coloration and fluorescent cocoon formation [10,20,21,22,25,26,27,29]. Therefore, we detected the total flavonoid contents in the sex-dependent fluorescent silkworm cocoons and found that the total flavonoid contents were comparable, suggesting that the different fluorescent phenotypes may be attributed to specific flavonoid subclasses or individual flavonoid compounds.

Furthermore, targeted metabolomics on flavonoids was employed to analyze the flavonoid profiles in the sex-dependent fluorescent silkworm cocoons. The results showed that flavones and flavonols constituted the predominant proportion of total flavonoids in both violet and yellow fluorescent cocoon types, consistent with previous reports of their high abundance in plants [17,38]. Notably, yellow cocoons contained approximately twice the level of flavonols but only half the level of other flavonoids compared to violet cocoons, highlighting that modified flavonoids may contribute to the divergent fluorescent phenotypes [10]. Similarly, numerous studies have reported an association between flavonoid metabolism and pigmentation in other species [39,40,41]. Previous studies have demonstrated that quercetin and its metabolites play critical roles in cocoon pigmentation and fluorescence [20,22,27]. Consistently, quercetin 3,7-diglucoside was identified as a key candidate marker associated with the formation of fluorescent silkworm cocoons in the present study, which was enriched in yellow cocoons. Also, quercetin 3,7-diglucoside was the most abundant cocoon flavonoid. However, it remained unclear whether it was derived directly from the diet or resulted from sex-specific differences in the absorption and metabolism of quercetin. Given that differential expression of genes involved in quercetin metabolism has been implicated in pigmentation, integrated metabolomic and transcriptomic analyses are needed to further reveal the potential molecular basis underlying the observed sex-dependent fluorescence [20,21,22,25,26]. Additionally, three other differential flavonoids with relatively lower abundances (baimaside, luteolin-3′,7-di-O-glucoside, and spiraeoside) may also contribute to the formation of the sex-dependent fluorescent phenotypes, although no direct evidence linking these compounds to fluorescence has been reported to date. Nevertheless, our findings further support this established knowledge and align with the known genetic framework underlying flavonoid-mediated cocoon coloration [10,20,21,22,25,27,28,42]. It has also been reported that fluorescent pigments in silkworm tissues may associate with pigment-binding protein [29]. However, to date, the mechanisms underlying fluorescence formation in the sex-discriminating fluorescent cocoons remain poorly understood. Our findings thus highlighted a valuable avenue for future research.

In conclusion, this was the first report on flavonoid profiles within sex-dependent fluorescent silkworm cocoons based on targeted metabolomics. Our results revealed that while the total flavonoid content was comparable between yellow (male) and violet (female) fluorescent cocoons, marked differences were observed in both the composition and abundance of individual flavonoid subclasses. Notably, quercetin 3,7-diglucoside may serve as the most promising biomarker associated with the sex-dependent fluorescence of silkworm cocoons. These findings not only provided a foundation for elucidating the mechanisms underlying fluorescence formation but also offered theoretical support for the breeding of sex-discriminating fluorescent cocoons and the development of methods for producing high-quality silk.

## Figures and Tables

**Table 1:** The number of flavonoid species, contents, and indicated percentage of each subclass in sex-dependent fluorescent silkworm cocoons.

Flavonoid Classes	Number of Species	Levels (µg/g Violet Cocoon) Mean ± SEM	Levels (µg/g Yellow Cocoon) Mean ± SEM	Percentage (%) Violet Cocoon	Percentage (%) Yellow Cocoon
Flavones	28	2.7177 ± 0.6135	7.8965 ± 0.4276	1.5714	2.6412
Isoflavones	11	0.2629 ± 0.0304	0.2528 ± 0.0161	0.1520	0.0846
Anthocyanidins	1	0.0187 ± 0.0075	0.0189 ± 0.0065	0.0108	0.0063
Flavonols	32	57.9065 ± 19.7538	193.8706 ± 24.5668	33.4811	61.5006
Flavanones	12	1.8428 ± 0.1022	2.4538 ± 0.1326	1.0655	0.8207
Flavanols	10	2.6180 ± 0.1091	2.7215 ± 0.2523	1.5137	0.9103
Chalcones	10	0.8908 ± 0.7798	4.2470 ± 2.8104	0.5150	1.4205
Other flavonoids	48	106.6942 ± 20.3310	97.5125 ± 19.4813	61.6906	32.6158

## Data Availability

The raw data supporting the conclusions of this article will be made available by the authors on request.

## References

[1] Federico G., Federico G. (1997). The roots of growth: Agricultural production. An Economic History of the Silk Industry, 1830–1930.

[2] Monir S., Biswas M.K., Hossain M.S. (2026). Globalization in Silk Production: A Comprehensive Review. Asian J. Agric. Ext. Econ. Sociol..

[3] Suárez-Sánchez M.F., Merritt H., Muñoz-Ruiz C.V., Suárez-Sánchez M., Oregel-Zamudio E., Arias-Martínez S. (2025). Frugal Innovation and Patent Analysis in Sericulture: Lessons for Sustainable Rural Bioeconomy Systems. Sustainability.

[4] Huang W., Ling S., Li C., Omenetto F.G., Kaplan D.L. (2018). Silkworm silk-based materials and devices generated using bio-nanotechnology. Chem. Soc. Rev..

[5] Aigner T.B., DeSimone E., Scheibel T. (2018). Biomedical Applications of Recombinant Silk-Based Materials. Adv. Mater..

[6] Johnson W., Bergfeld W.F., Belsito D.V., Hill R.A., Klaassen C.D., Liebler D.C., Marks J.G., Shank R.C., Slaga T.J., Snyder P.W. (2020). Safety Assessment of Silk Protein Ingredients as Used in Cosmetics. Int. J. Toxicol..

[7] Aad R., Dragojlov I., Vesentini S. (2024). Sericin Protein: Structure, Properties, and Applications. J. Funct. Biomater..

[8] Guncheva R. (2023). Sex ratio of the pupal stage in cocoons with different fluorescence characteristics from silkworm *Bombyx mori L*. breeds and hybrids. Bulg. J. Agric. Sci..

[9] Guncheva R. (2023). Study of the relationship between fluorescence type and percentage content of good-quality cocoons of the silkworm *Bombyx mori L*. breeds and hybrids. Bulg. J. Agric. Sci..

[10] Zhang Y., Yu X., Shen W., Ma Y., Zhou L., Xu N., Yi S. (2010). Mechanism of fluorescent cocoon sex identification for silkworms *Bombyx mori*. Sci. China Life Sci..

[11] Ma G., Chai X., Hou G., Zhao F., Meng Q. (2022). Phytochemistry, bioactivities and future prospects of mulberry leaves: A review. Food Chem..

[12] Ju W.T., Kwon O.C., Kim H.B., Sung G.B., Kim H.W., Kim Y.S. (2018). Qualitative and quantitative analysis of flavonoids from 12 species of Korean mulberry leaves. J. Food Sci. Technol..

[13] Thaipitakwong T., Numhom S., Aramwit P. (2018). Mulberry leaves and their potential effects against cardiometabolic risks: A review of chemical compositions, biological properties and clinical efficacy. Pharm. Biol..

[14] Williamson G., Kay C.D., Crozier A. (2018). The Bioavailability, Transport, and Bioactivity of Dietary Flavonoids: A Review from a Historical Perspective. Compr. Rev. Food Sci. Food Saf..

[15] Galleano M., Verstraeten S.V., Oteiza P.I., Fraga C.G. (2010). Antioxidant actions of flavonoids: Thermodynamic and kinetic analysis. Arch. Biochem. Biophys..

[16] Homayoonfal M., Aminianfar A., Asemi Z., Yousefi B. (2024). Application of Nanoparticles for Efficient Delivery of Quercetin in Cancer Cells. Curr. Med. Chem..

[17] Shen N., Wang T., Gan Q., Liu S., Wang L., Jin B. (2022). Plant flavonoids: Classification, distribution, biosynthesis, and antioxidant activity. Food Chem..

[18] Vissenaekens H., Criel H., Grootaert C., Raes K., Smagghe G., Van Camp J. (2022). Flavonoids and cellular stress: A complex interplay affecting human health. Crit. Rev. Food Sci. Nutr..

[19] Gentile D., Fornai M., Pellegrini C., Colucci R., Blandizzi C., Antonioli L. (2018). Dietary flavonoids as a potential intervention to improve redox balance in obesity and related co-morbidities: A review. Nutr. Res. Rev..

[20] Daimon T., Hirayama C., Kanai M., Ruike Y., Meng Y., Kosegawa E., Nakamura M., Tsujimoto G., Katsuma S., Shimada T. (2010). The silkworm Green b locus encodes a quercetin 5-O-glucosyltransferase that produces green cocoons with UV-shielding properties. Proc. Natl. Acad. Sci. USA.

[21] Lu Y., Luo J., An E., Lu B., Wei Y., Chen X., Lu K., Liang S., Hu H., Han M. (2023). Deciphering the Genetic Basis of Silkworm Cocoon Colors Provides New Insights into Biological Coloration and Phenotypic Diversification. Mol. Biol. Evol..

[22] Xu X., Wang M., Wang Y., Sima Y., Zhang D., Li J., Yin W., Xu S. (2013). Green cocoons in silkworm *Bombyx mori* resulting from the quercetin 5-O-glucosyltransferase of UGT86, is an evolved response to dietary toxins. Mol. Biol. Rep..

[23] Albert N.W., Davies K.M., Schwinn K.E. (2014). Gene regulation networks generate diverse pigmentation patterns in plants. Plant Signal Behav..

[24] Jiang L., Yang X., Gao X., Yang H., Ma S., Huang S., Zhu J., Zhou H., Li X., Gu X. (2024). Multiomics Analyses Reveal the Dual Role of Flavonoids in Pigmentation and Abiotic Stress Tolerance of Soybean Seeds. J. Agric. Food Chem..

[25] Hirayama C., Mase K., Iizuka T., Takasu Y., Okada E., Yamamoto K. (2018). Deficiency of a pyrroline-5-carboxylate reductase produces the yellowish green cocoon ‘Ryokuken’ of the silkworm, *Bombyx mori*. Heredity.

[26] Waizumi R., Hirayama C., Tomita S., Iizuka T., Kuwazaki S., Jouraku A., Tsubota T., Yokoi K., Yamamoto K., Sezutsu H. (2024). A major endogenous glycoside hydrolase mediating quercetin uptake in *Bombyx mori*. PLoS Genet..

[27] Kusurkar T.S., Tandon I., Sethy N.K., Bhargava K., Sarkar S., Singh S.K., Das M. (2013). Fluorescent silk cocoon creating fluorescent diatom using a “Water glass-fluorophore ferry”. Sci. Rep..

[28] Zhang Y., Dong Z., Zhao D., Li H., Wang L., Lin Y., Zhao P. (2019). Comparison of chemical constituents of wild silkworm cocoon and domestic silkworm cocoon by UHPLC-MS technology. Sheng Wu Gong Cheng Xue Bao.

[29] Hu X., Xue R., Cao G., Zhang X., Zhang Y., Yu X., Zhang Y., Gong C. (2012). Elementary research of the formation mechanism of sex-related fluorescent cocoon of silkworm, *Bombyx mori*. Mol. Biol. Rep..

[30] Tong C.Y., Shi F.Y., Tong X., Shi S.Y., Ali I., Guo Y. (2021). Shining natural flavonols in sensing and bioimaging. Trac-Trend Anal. Chem..

[31] Li H., Li M., Zhang F., Nie L. (2026). Pigment analysis of Sex-Specific fluorescent cocoon coloration in *Bombyx mori* strain Chunyu. J. Asia-Pac. Entomol..

[32] Deo S.K., Daunert S. (2001). Luminescent proteins from Aequorea victoria: Applications in drug discovery and in high throughput analysis. Fresenius J. Anal. Chem..

[33] Yoda S., Sakakura K., Kitamura T., KonDo Y., Sato K., Ohnuki R., Someya I., Komata S., Kojima T., Yoshioka S. (2021). Genetic switch in UV response of mimicry-related pale-yellow colors in Batesian mimic butterfly, Papilio polytes. Sci. Adv..

[34] Daney de Marcillac W., Nguyen L.T.P., Aracheloff C., Berthier S., Schollhorn B. (2021). Bright green fluorescence of Asian paper wasp nests. J. R. Soc. Interface.

[35] Joseph Raj A.N., Sundaram R., Mahesh V.G.V., Zhuang Z., Simeone A. (2019). A Multi-Sensor System for Silkworm Cocoon Gender Classification via Image Processing and Support Vector Machine. Sensors.

[36] Wu Z.F., Wang B.J., Ni J.W., Sun Z.N., Zhang X.R., Xiong H.M. (2024). Green Fluorescent Carbon Dots with Critically Controlled Surface States: Make Silk Shine via Feeding Silkworms. Nano Lett..

[37] Liu J., Kong T., Xiong H.M. (2022). Mulberry-Leaves-Derived Red-Emissive Carbon Dots for Feeding Silkworms to Produce Brightly Fluorescent Silk. Adv. Mater..

[38] Li D., Chen G., Ma B., Zhong C., He N. (2020). Metabolic Profiling and Transcriptome Analysis of Mulberry Leaves Provide Insights into Flavonoid Biosynthesis. J. Agric. Food Chem..

[39] Hossain M.N., Sarker U., Raihan M.S., Al-Huqail A.A., Siddiqui M.H., Oba S. (2022). Influence of Salinity Stress on Color Parameters, Leaf Pigmentation, Polyphenol and Flavonoid Contents, and Antioxidant Activity of Amaranthus lividus Leafy Vegetables. Molecules.

[40] Ryu J.A., Duan S., Jeong H.Y., Lee C., Kang I.K., Eom S.H. (2022). Pigmentation and Flavonoid Metabolite Diversity in Immature ‘Fuji’ Apple Fruits in Response to Lights and Methyl Jasmonate. Int. J. Mol. Sci..

[41] Espley R.V., Jaakola L. (2023). The role of environmental stress in fruit pigmentation. Plant Cell Environ..

[42] Mase K., Hirayama C., Narukawa J., Kuwazaki S., Yamamoto K. (2023). Fine mapping of Green a, Ga, on chromosome 27 in *Bombyx mori*. Genes Genet. Syst..

